# Identification of an immune-related genes signature in lung adenocarcinoma to predict survival and response to immune checkpoint inhibitors

**DOI:** 10.1186/s43046-024-00236-0

**Published:** 2024-10-07

**Authors:** Zeinab Davoodi-Moghaddam, Farideh Jafari-Raddani, Shahram Kordasti, Davood Bashash

**Affiliations:** 1 Department of Hematology and Blood Banking, School of Allied Medical Sciences, Shahid Beheshti University of Medical Sciences, Tehran, Iran; 2 Comprehensive Cancer Centre, School of Cancer and Pharmaceutical Sciences, Faculty of Life Sciences and Medicine, King’s College London, London, UK; 3 Haematology Department, Guy’s Hospital, London, UK

**Keywords:** Lung adenocarcinoma, Immune-related signature, Immunotherapy, Immune checkpoint inhibitor, Tumor immune microenvironment

## Abstract

**Background:**

Although advances in immune checkpoint inhibitor (ICI) research have provided a new treatment approach for lung adenocarcinoma (LUAD) patients, their survival is still unsatisfactory, and there are issues in the era of response prediction to immunotherapy.

**Methods:**

Using bioinformatics methods, a prognostic signature was constructed, and its predictive ability was validated both in the internal and external datasets (GSE68465). We also explored the tumor-infiltrating immune cells, mutation profiles, and immunophenoscore (IPS) in the low-and high-risk groups.

**Results:**

As far as we are aware, this is the first study which introduces a novel prognostic signature model using BIRC5, CBLC, S100P, SHC3, ANOS1, VIPR1, LGR4, PGC, and IGKV4.1. According to multivariate analysis, the 9-immune-related genes (IRGs) signature provided an independent prognostic factor for the overall survival (OS). The low-risk group had better OS, and the tumor mutation burden (TMB) was significantly lower in this group. Moreover, the risk scores were negatively associated with the tumor-infiltrating immune cells, like CD8^+^ T cells, macrophages, dendritic cells, and NK cells. In addition, the IPS were significantly higher in the low-risk group as they had higher gene expression of immune checkpoints, suggesting that ICIs could be a promising treatment option for low-risk LUAD patients.

**Conclusion:**

The combination of these 9-IRGs not only could efficiently predict overall survival of LUAD patients but also show a powerful association with the expression of immune checkpoints and response to ICIs based on IPS; hoping this model paves the way for better stratification and management of patients in clinical practice.

**Supplementary Information:**

The online version contains supplementary material available at 10.1186/s43046-024-00236-0.

## Introduction

Lung cancer stood out as the foremost contributor to cancer-related mortality and the second most frequently occurring cancer in 2020, accounting for one in five (18.0%) cancer deaths and one in ten (11.4%) cancers diagnosed [1]. Non-small-cell lung cancer (NSCLC) represents about 85% of all lung cancer cases, with lung adenocarcinoma (LUAD) emerging as the most prevalent subtype diagnosed in non-smokers [2, 3]. Currently, surgical resection, as well as other standard treatments, have increased the survival rates of patients with localized and early-stage cancer, whereas most LUAD patients with advanced disease experience high mortality rates [4]. In recent years, immune checkpoint (IC) inhibition using anti-PD1 or anti-PD-L1 antibodies has demonstrated striking clinical responses in NSCLC patients; However, it is worth noting that only a specific subgroup of patients experiences lasting clinical advantages [5–7]. There is evidence that biomarker-driven treatment can improve survival rates in advanced and metastatic LUAD [8–10]; therefore, identifying and developing biomarkers to predict the responsiveness of checkpoint inhibitor-based immunotherapy is crucial for a more effective approach to cancer immunotherapy.

The tumor immune microenvironment (TIME) contains immune cells, inflammatory mediators, endothelial cells, mesenchymal cells, and extracellular matrix (ECM) molecules [11]. The density, location, and type of immune cells in TIME influence the disease progression and could be a promising new approach as predictive biomarkers for corresponding cancer prognosis [12]. Moreover, a growing body of evidence indicates that TIME plays a vital role in anti-cancer immunity, which may result in resistance to immune checkpoint inhibitor therapy [13–15]. This study aimed to develop a signature based on immune-related genes which could predict the prognosis and response to ICI treatment in patients with LUAD. Following the construction of the model, its relationship to prognosis and clinicopathological characteristics was investigated in The Cancer Genome Atlas Lung Adenocarcinoma (TCGA-LUAD) cohort. Furthermore, we explored the tumor-infiltrating immune cells, mutation profiles, and immunophenoscore (IPS) related to this signature in LUAD. This may be implemented to predict the overall survival and thereby improve future ICI treatment for LUAD.

## Methods

### Data collection

We downloaded the LUAD patients’ transcription profiles and clinical data from Cancer Genome Atlas (TCGA) data portal (https://portal.gdc.cancer.gov/) using the R package “TCGAbiolinks.” We also downloaded another microarray dataset (GSE68465) from Gene Expression Omnibus (GEO) database (https://www.ncbi.nlm.nih.gov/geo/) for further validation of the signature. The inclusive list of IRGs was obtained from the Immunology Database and Analysis Portal (ImmPort) database (https://immport.niaid.nih.gov) [16]. The immunophenoscore of patients was gained from The Cancer Immunome Atlas (TCIA) database (https://tcia.at/home).

### Screening of DEGs

After normalization of the TCGA dataset, to identify the IRGs which contributed to LUAD progression, differentially expressed genes (DEGs) between tumor and normal samples were screened using the “limma” package [17]. We set the significance criteria as follows:| logFC |> 2 and adjusted *P*-value < 0.01. After intersecting IRGs from the ImmPort database, we identified differentially expressed immune-related genes (DE IRGs).

### Functional enrichment analyses

The Gene Ontology (GO) and Kyoto Encyclopedia of Genes and Genomes (KEGG) enrichment analyses were used to explore the possible molecular mechanisms of DE IRGs using the “clusterProfiler” R package [18]. Adjusted *P-value* below 0.05 was deemed to be statistically significant. To get much more information, we also used the ToppFun enrichment (https://toppgene.cchmc.org/enrichment.jsp).

### Construction of prognostic prediction model based on risk score

At this step, normal samples and samples without survival data were excluded, and the rest of the TCGA-LUAD project underwent a random division into training and testing cohorts. The training set was utilized for the identification of prognostic IRGs and the establishment of a prognostic immune-related risk model, while prognostic qualification was validated using the testing cohort. To pinpoint potential DE IRGs with prognostic value, identified DE IRGs were subjected to univariate Cox regression analysis using “survminer” and “survival” R packages. Then a least absolute shrinkage and selection operator (LASSO) penalized Cox proportional hazards regression was conducted on prognosis-related DE IRGs to find the best genes for constructing the model and minimize overfitting using the “glmnet” R package [19, 20]. Finally, the risk score of each LUAD patient was calculated based on gene expression and the corresponding multivariate Cox regression coefficient. The formula was as follows:

$$\text{Risk score}\hspace{0.17em}=\hspace{0.17em}(\text{expression of Gene }1\hspace{0.17em}\times \hspace{0.17em}\text{coefficient Gene }1)\hspace{0.17em}+\hspace{0.17em}(\text{expression of Gene }2\hspace{0.17em}\times \hspace{0.17em}\text{coefficient Gene }2)\hspace{0.17em}+\hspace{0.17em}\dots (\text{expression of Gene n}\hspace{0.17em}\times \hspace{0.17em}\text{coefficient Gene n})$$

### Evaluation of the established immune-related signature

Based on the median cutoff of risk score, patients were divided into low- and high-risk groups. To assess the prognostic value of the DE IRG model, the Kaplan–Meier analysis was performed using “survminer” and “survival” R packages. To evaluate the sensitivity and specificity of the immune-related risk signature, the receiver operating characteristic (ROC) curve analyses of 1-, 3-, and 5-year were used, and the area under the curve (AUC) was calculated using “survivalROC” R package [21]. Univariate and multivariate Cox regression analyses were employed to assess the independent prognostic value of risk score and clinicopathological features, such as age, gender, TNM stage, and clinical stage. In addition, we used the Wilcoxon test to determine the differences between the clinicopathological characteristics of patients in terms of risk scores.

### Investigation of tumor-infiltrating immune cells

Various methods for estimating immune infiltration, including CIBERSORT, quanTIseq, TIMER, and XCELL were used to investigate the status of immune infiltration among LUAD patients. An analysis of the Spearman correlation was conducted to determine the relationship between immune infiltrating cells and risk scores.

### Mutation analysis

Mutation data in the form of Mutation Annotation Format (MAF) and tumor mutation burden (TMB) was obtained from the TCGA portal, and the “maftools” R package was used to analyze it [22].

### Immunophenoscore analysis

The immunogenicity is assessed by MHC molecules, immunosuppressive cells, effector cells, and immunomodulators that collectively make up four significant categories of genes, from which machine learning can determine the patient’s IPS without bias. IPS is calculated using a scale from 0 to 10, with higher scores representing a greater level of immunogenicity [23]. The IPS results of 20 different solid tumors can be accessed at (https://tcia.at/home).

### Statistical analysis

**Fig. 1:**
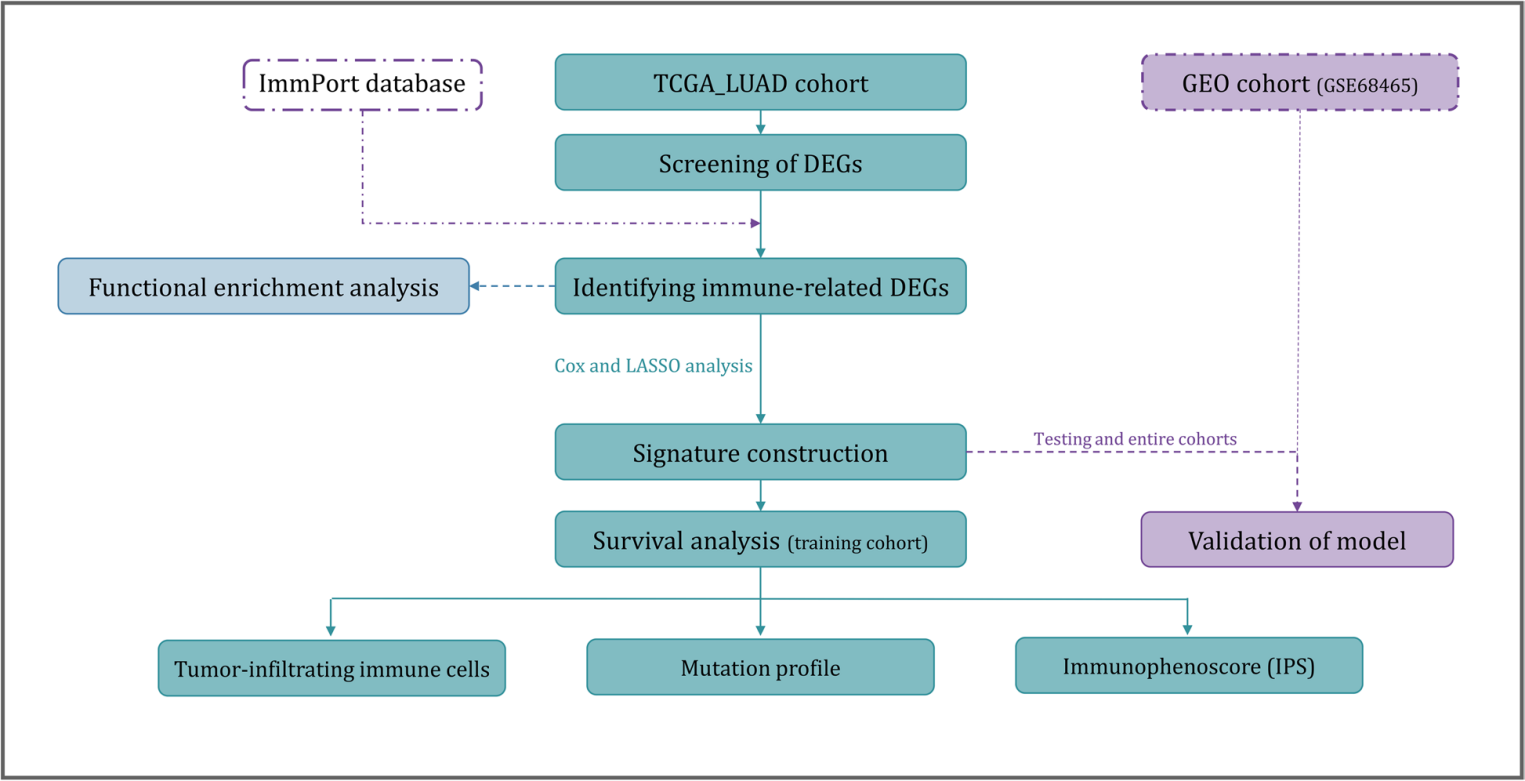
The flowchart of the study

The statistical analyses were conducted using R software version 4.2.1 and GraphPad Prism version 9.4. We used the R package “pheatmap” to create the heatmap and the package “ggplot2” to generate the volcano plot. A Venn diagram was generated on the site of (https://bioinformatics.psb.ugent.be/webtools/Venn). The flowchart of the study is depicted in Fig. 1.

## Results

### Patients’ characteristics

Among the 598 samples analyzed in the TCGA-LUAD project, 13 patients had no clinical and survival data. Therefore, RNA-sequencing expression profiles and other information from 59 normal and 526 LUAD samples were included in this study. The LUAD samples were randomly split into two groups: a training cohort with 421 samples and a testing cohort with 105 samples. Table S1 details the clinical characteristics of samples in the training, testing, and entire cohorts, indicating no significant differences among them (*P* > 0.05).

### Screening of DE-IRGs

**Fig. 2:**
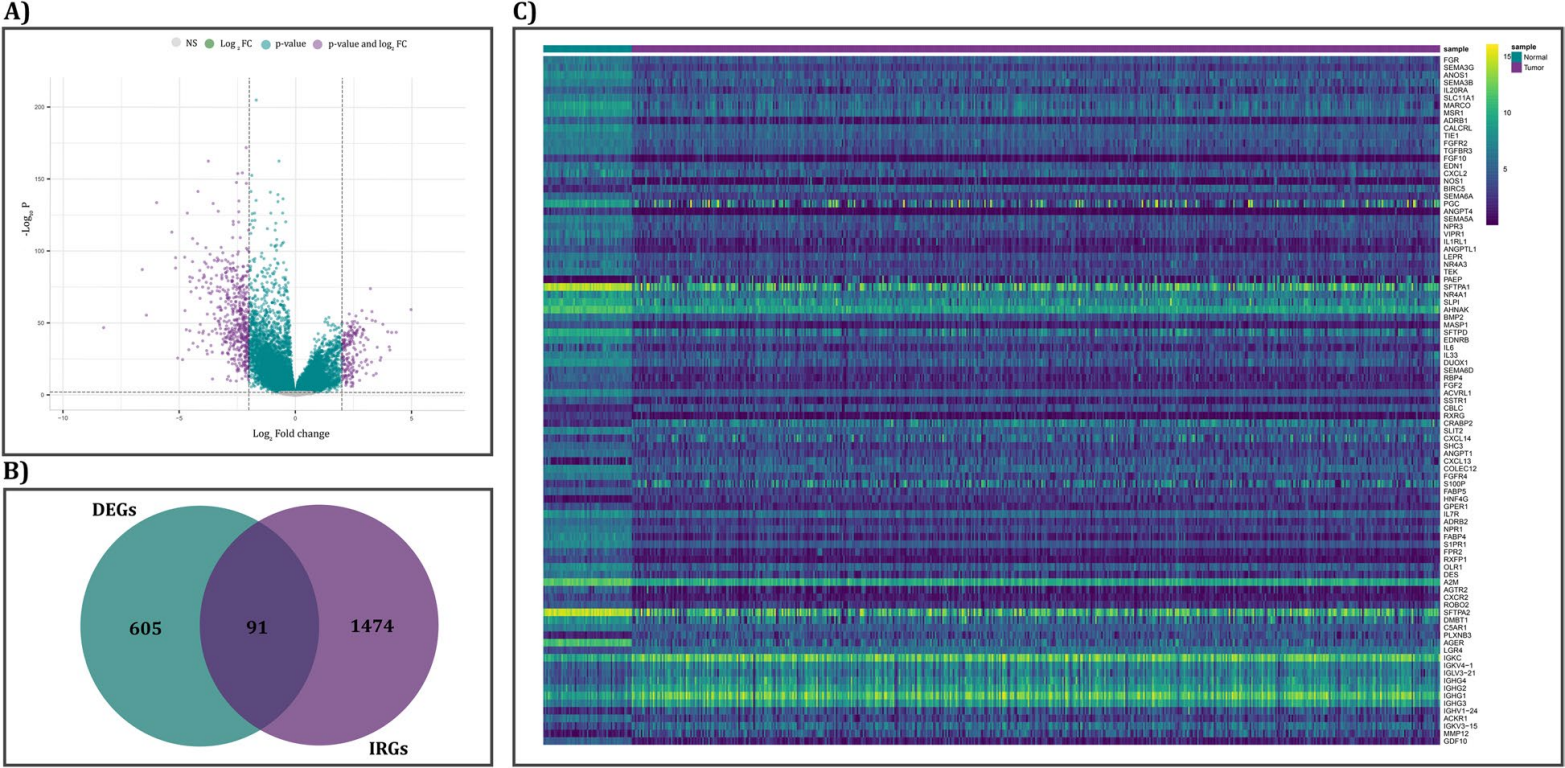
Identification of DE-IRGs between LUAD samples and normal samples.** A** Volcano plot of DEGs based on TCGA-LUAD project. **B** Venn diagram for the intersections between LUAD DEGs and IRGs. **C** The heatmap of DE-IRGs expression between the normal and tumor samples

According to the adjusted *P*-value < 0.01 and |log2 (fold change)|> 2, a total of 696 DEGs were identified between normal and LUAD samples of the TCGA-LUAD project for further analysis (Fig. 2A). After integrating 1565 IRGs, we obtained 91 DE-IRGs (Fig. 2B), of which 20 DE-IRGs were up-regulated, and 71 DE-IRGs were downregulated (Table S2). DE-IRGs expression profile of normal and tumor samples is shown in Fig. 2C.

### Functional enrichment analysis

**Fig. 3:**
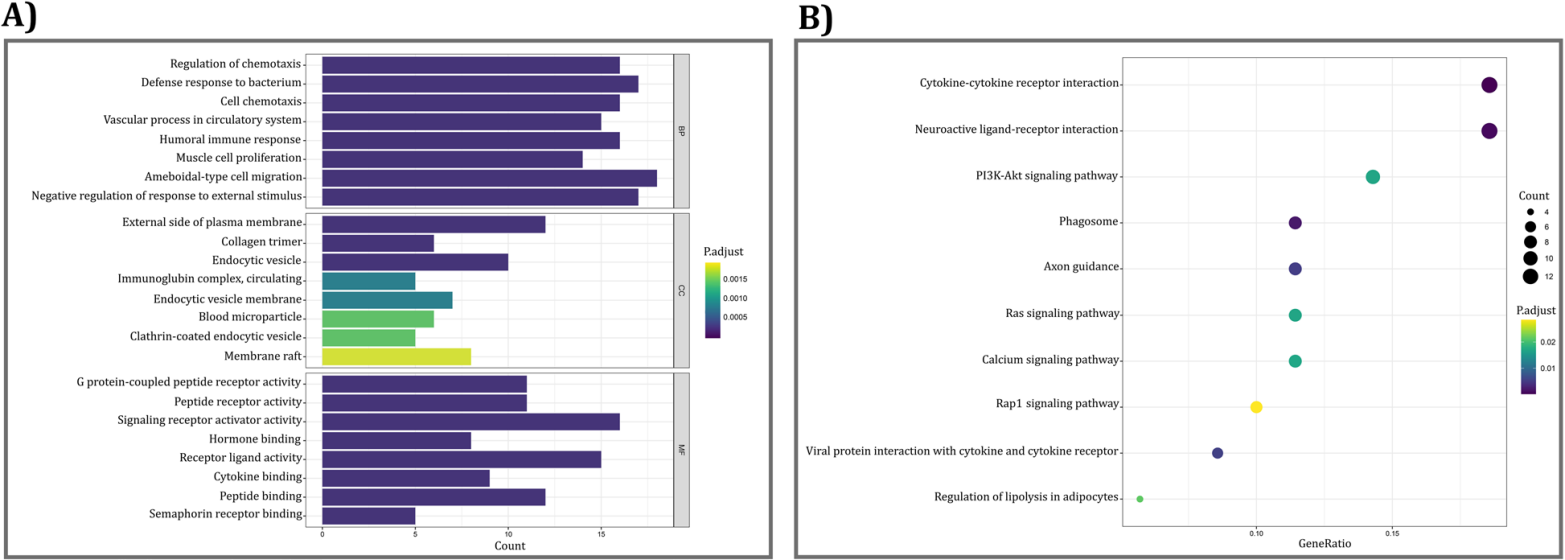
Functional enrichment analyses of DE-IRGs.** A** Most significant enriched Gene ontology (GO) categories for the validated DE-IRGs. **B** The enriched pathways of the DE-IRGs

To better understand the underlying mechanisms and predict the prognosis of LUAD, we further investigated the functions and pathways affected by these 91 DE-IRGs. GO analysis indicated that the most significantly (adjusted *P*-value < 0.05) enriched terms for biological process, molecular function, and cellular component were “regulation of chemotaxis,” “G protein-coupled peptide receptor activity,” and “external side of plasma membrane,” respectively. The most ten highly enriched terms for the three ontologies are represented in Fig. 3A and Table 1. To identify possible signaling pathways associated with DE-IRGs, we conducted an analysis of KEGG with data from the TCGA cohort (Fig. 3B and Table 2). The results of ToppFun enrichment are also summarized in Table S3.

**Table 1:** The list of 10 most significant enriched GO categories for DE-IRGs. (Adjusted *P*-value < 0.05)

**IDs**	**Term**	**Adj. *P*-value**	**Count**
**Biological process**			
GO:0050920	Regulation of chemotaxis	1.47E-11	16
GO:0042742	Defense response to bacterium	5.99E-10	17
GO:0060326	Cell chemotaxis	5.99E-10	16
GO:0003018	Vascular process in circulatory system	5.99E-10	15
GO:0006959	Humoral immune response	5.99E-10	16
GO:0033002	Muscle cell proliferation	1.4E-09	14
GO:0001667	Ameboidal-type cell migration	2.13E-09	18
GO:0032102	Negative regulation of response to external stimulus	3.27E-09	17
GO:0050673	Epithelial cell proliferation	4.83E-09	17
GO:0050921	Positive regulation of chemotaxis	1.15E-08	11
**Cellular component**			
GO:0009897	External side of plasma membrane	0.000119	12
GO:0005581	Collagen trimer	0.000143	6
GO:0030139	Endocytic vesicle	0.000143	10
GO:0042571	Immunoglobulin complex, circulating	0.000767	5
GO:0030666	Endocytic vesicle membrane	0.000767	7
GO:0072562	Blood microparticle	0.001157	6
GO:0045334	Clathrin-coated endocytic vesicle	0.001157	5
GO:0045121	Membrane raft	0.001747	8
GO:0098857	Membrane microdomain	0.001747	8
GO:0062023	Collagen-containing extracellular matrix	0.001845	9
**Molecular function**			
GO:0008528	G protein-coupled peptide receptor activity	1.81E-08	11
GO:0001653	Peptide receptor activity	1.81E-08	11
GO:0030546	Signaling receptor activator activity	9.38E-08	16
GO:0042562	Hormone binding	3.87E-07	8
GO:0048018	Receptor ligand activity	3.87E-07	15
GO:0019955	Cytokine binding	8.24E-07	9
GO:0042277	Peptide binding	1.19E-06	12
GO:0030215	Semaphorin receptor binding	1.88E-06	5
GO:0038024	Cargo receptor activity	2.3E-06	7
GO:0017046	Peptide hormone binding	3.65E-06	6

**Table 2:** The list of most significantly enriched pathways for DE-IRGs. (Adj* P*-value < 0.05)

**No.**	**Pathway IDs**	**Pathway names**	**Adj. *P*-value**	**Count**
1	hsa04060	Cytokine-cytokine receptor interaction	0.000143	13
2	hsa04080	Neuroactive ligand-receptor interaction	0.000686	13
3	hsa04145	Phagosome	0.001978	8
4	hsa04360	Axon guidance	0.005275	8
5	hsa04061	Viral protein interaction with cytokine and cytokine receptor	0.005625	6
6	hsa04151	PI3K-Akt signaling pathway	0.016834	10
7	hsa04014	Ras signaling pathway	0.016834	8
8	hsa04020	Calcium signaling pathway	0.016892	8
9	hsa04923	Regulation of lipolysis in adipocytes	0.021483	4
10	hsa04015	Rap1 signaling pathway	0.028469	7
11	hsa04066	HIF-1 signaling pathway	0.029728	5
12	hsa04924	Renin secretion	0.032646	4
13	hsa04010	MAPK signaling pathway	0.037333	8
14	hsa03320	PPAR signaling pathway	0.037858	4
15	hsa01521	EGFR tyrosine kinase inhibitor resistance	0.042211	4
16	hsa04926	Relaxin signaling pathway	0.042211	5
17	hsa04062	Chemokine signaling pathway	0.048183	6

### Construction of prognostic prediction model based on risk score

**Fig. 4:**
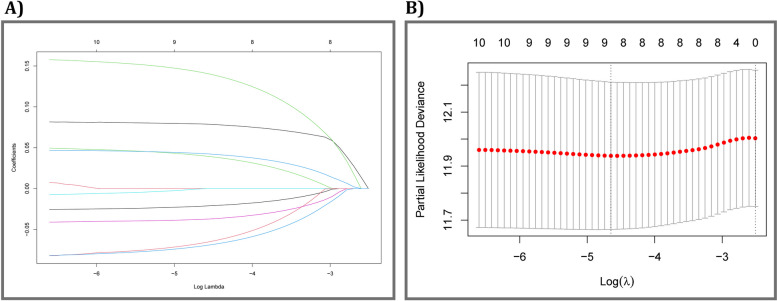
Construction of prognostic prediction signature. LASSO regression was performed to identify the minimum criteria

**Fig. 5:**
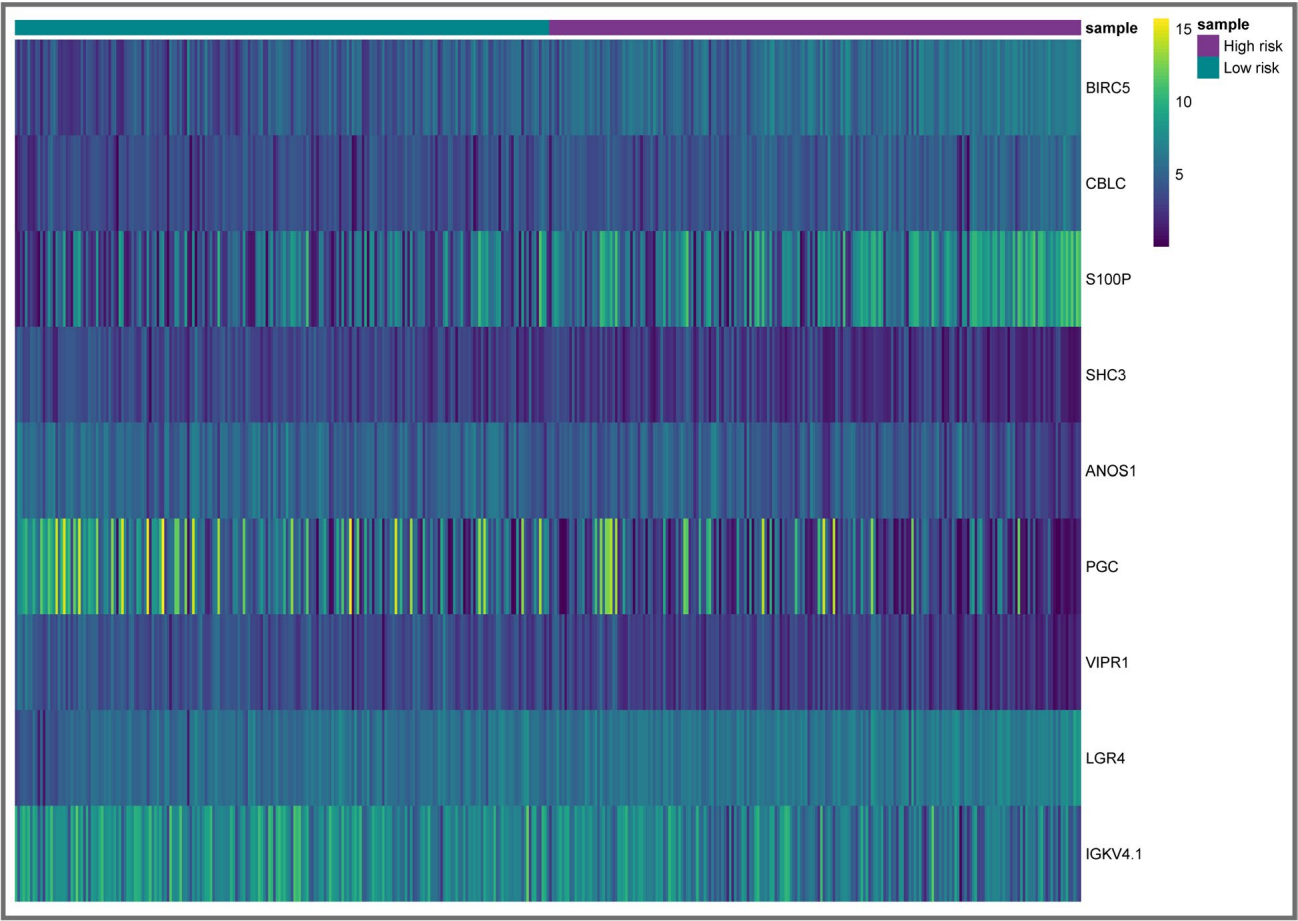
The heatmap of 9 DE- IRGs between two risk groups in the total cohort

A univariate Cox regression analysis was performed to recognize potentially predictive genes among DE-IRGs, and 10 DE-IRGs were found to have significant relations to OS (*P* < 0.05) in the training cohort of LUAD patients (Table 3). Next, the candidate genes underwent LASSO Cox regression analysis to eliminate genes with high correlations and minimize overfitting. Totally, 9 of the 10 DE-IRGs were screened (Fig. 4). The heatmap of these 9 DE- IRGs between two risk groups in the entire cohort is depicted in Fig. 5. We utilized these 9 DE-IRGs to construct the prognosis predictive model by multivariate Cox regression analysis (Table 4) and calculated the risk score as follows:

**Table 3:** Univariate cox

	**Gene**	**Coef.**	**HR**	**HR.95L**	**HR.95H**	***P*-value**
1	BIRC5	0.16325	1.177	1.048	1.322	0.00588^**^
2	GDF10	-0.16007	0.8521	0.7266	0.9992	0.0489^*^
3	CBLC	0.17262	1.188	1.033	1.367	0.0158^*^
4	S100P	0.06846	1.071	1.015	1.13	0.0127^*^
5	SHC3	-0.1382	0.8709	0.7636	0.9933	0.0394^*^
6	ANOS1	-0.14192	0.8677	0.7702	0.9776	0.0196^*^
7	PGC	-0.04348	0.9574	0.9168	0.9999	0.0496^*^
8	VIPR1	-0.15594	0.8556	0.7466	0.9805	0.0249^*^
9	LGR4	0.20812	1.231	1.05	1.444	0.0103^*^
10	IGKV4.1	-0.07662	0.9262	0.8611	0.9963	0.0395^*^

^*^indicates a *P*-value < 0.05; ^**^indicates a *P*-value < 0.001

**Table 4:** Coefficients and multivariable cox model results of 9 IRGs in risk signature

	**Gene**	**Coef.**	**HR**	**HR.95L**	**HR.95H**	***P*-value**
1	BIRC5	0.081428	1.0848	0.9309	1.2642	0.2969
2	CBLC	0.050255	1.0515	0.8942	1.2365	0.5433
3	S100P	0.047464	1.0486	0.9786	1.1237	0.1783
4	SHC3	-0.008143	0.9919	0.8444	1.1652	0.9211
5	ANOS1	-0.040922	0.9599	0.8281	1.1126	0.5870
6	PGC	-0.026052	0.9743	0.9246	1.0266	0.3289
7	VIPR1	-0.082896	0.9204	0.7782	1.0887	0.3330
8	LGR4	0.159715	1.1732	0.9926	1.3866	0.0611
9	IGKV4.1	-0.083087	0.9203	0.8515	0.9945	0.0359^*^

^*^indicates a *P*-value < 0.05

$$\mathrm{Risk}\;\mathrm{score}\:=\:(\mathrm{BIRC}5\;\exp.\:\times\:0.081428)\:+\:(\mathrm{CBLC}\;\exp.\:\times\:0.050255)\:+\:(\mathrm S100\mathrm P\;\exp.\:\times\:0.047464)\:+\:(\mathrm{SHC}3\;\exp.\:\times\:\:-\:0.008143)\:+\:(\mathrm{ANOS}1\;\exp.\:\times\:\:-\:0.040922)\:+\:(\mathrm{PGC}\;\exp.\:\times\:\:-\:0.026052)\:+\:(\mathrm{VIPR}1\;\exp.\:\times\:\:-\:0.082896)\:+\:(\mathrm{LGR}4\;\exp.\:\times\:0.159715)\:+\:(\mathrm{IGKV}4.1\;\exp.\:\times\:\:-\:0.083087)$$

### Validation of the prognostic prediction model

**Fig. 6:**
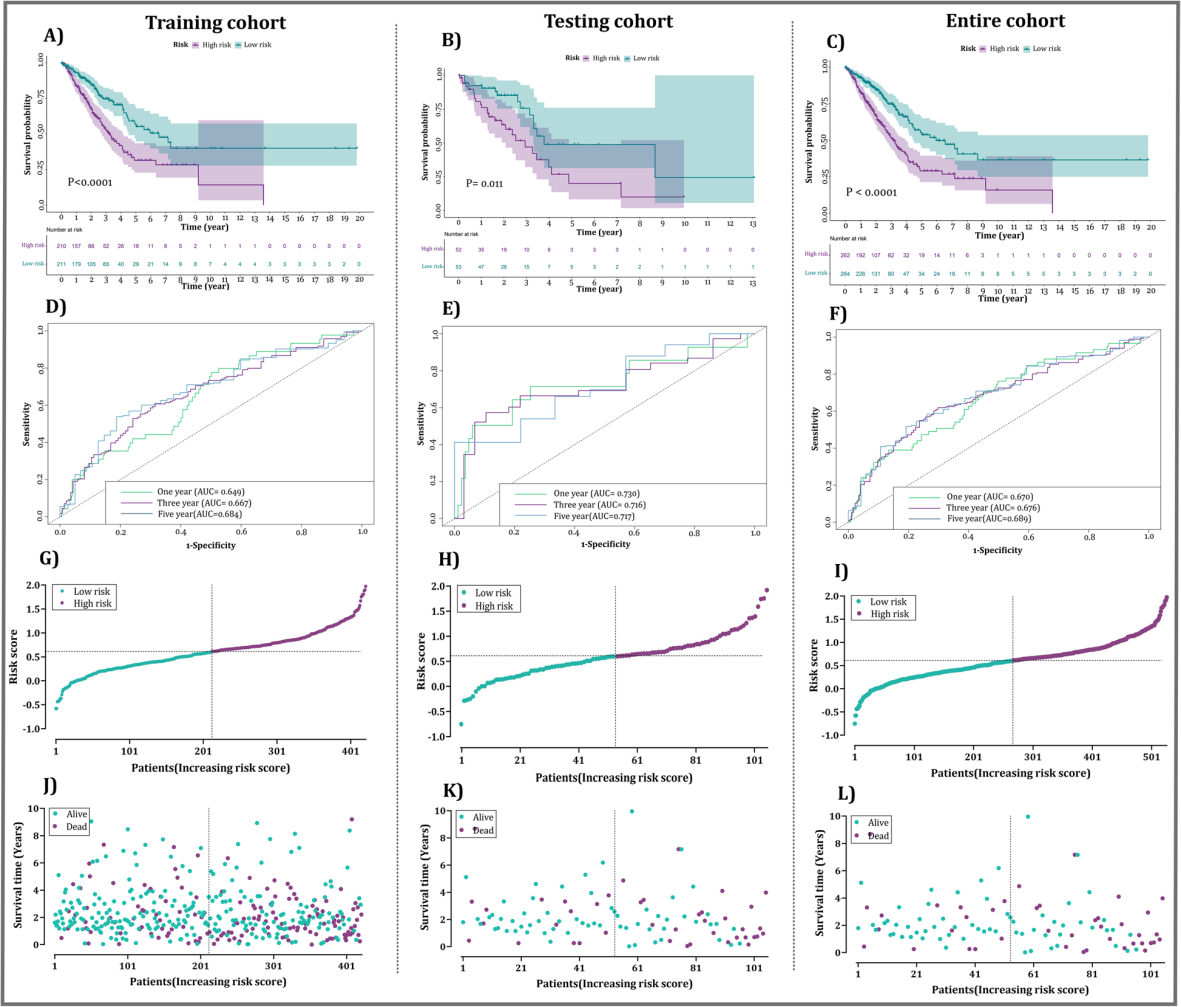
Validation of the immune-related signature in the TCGA cohort.** A**–**C** The Kaplan–Meier curve analysis of the high- and low-risk groups in the training, testing, and total cohorts. **D**–**F** ROC curve analysis of the prognostic prediction model in the training, testing, and entire cohorts. **G**–**L** The distribution of risk scores and survival status in the training, testing, and total cohorts

**Fig. 7:**
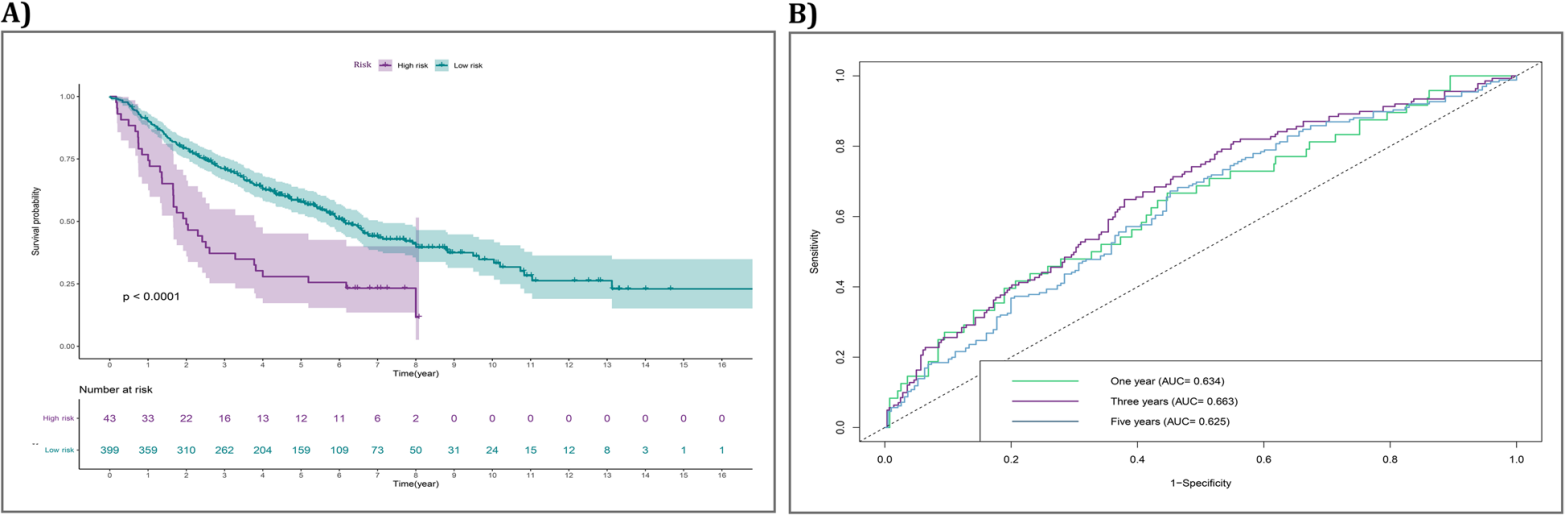
Validation of the immune-related signature in the GSE68465 cohort.** A** The Kaplan–Meier curve analysis of the high- and low-risk groups in GEO cohort. **B** ROC curve analysis of the prognostic prediction model in GEO cohort

To validate the immune-related gene signature, the entire testing cohorts were used as internal validation to verify its predictive capability. All LUAD patients within the three cohorts were stratified into low- and high-risk groups using the median risk score value derived from the training group. As the next step, we investigated how well the prognostic model could distinguish survival in patients’ risk groups. The analysis of Kaplan–Meier curves indicated a significant difference in OS among the two predicted groups of all cohorts, and high-risk patients had a poor outcome (Fig. 6A–C). The time-dependent ROC curves were performed to validate the accuracy of the model, and the 5-year AUC values gained 0.684, 0.717, and 0.689 in the training, testing, and entire cohorts, respectively (Fig. 6D–F), suggesting that it may be feasible to predict the survival of LUAD patients using this presented model. Additionally, the findings indicate that patients with higher risk scores are more prone to worse survival outcomes (Fig. 6G–L). Overall, these results showed satisfactory predictive performance of the IRGs signature in TCGA-LUAD data. We also validated our model using an external independent validation dataset (the GSE68465 dataset). Of note, the Kaplan–Meier curves analysis demonstrated a more satisfactory outcome for low-risk group patients. The results of Kaplan–Meier curves, as well as 1-, 3- and 5-year AUC, were represented in Fig. 7, further highlighting that the risk signature performed satisfactorily as a predictor of external data.

### Evaluation of the prognostic prediction model

#### Association between the model and clinicopathological features

**Fig. 8:**
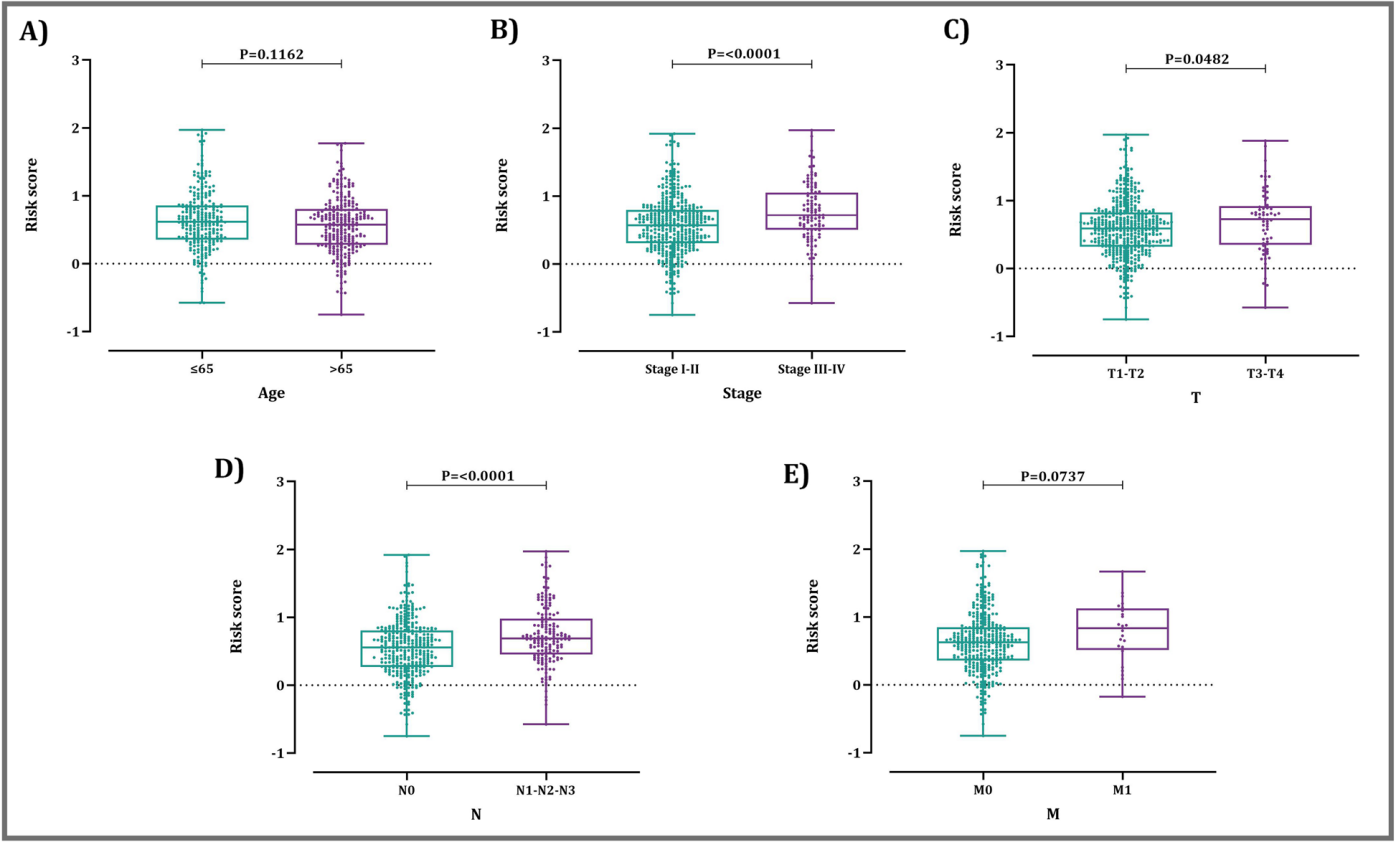
The relationships between the immune-related risk signature and **A** age; **B** clinical stage; **C** T stage; **D** N stage; **E** M stage

Interestingly, the results of the Wilcoxon rank sum test indicate a statistically significant association between the risk score and clinicopathological features of patients. Specifically, the 9-IRG risk score demonstrated a notably elevated correlation with advanced clinical T stage (*P* = 0.0482) and N stage (*P* < 0.0001) (Fig. 8C, D). Accordingly, the prognostic value of the model may partially be due to its association with clinicopathological characteristics.

#### Independent prognostic role of the model

Univariable Cox and multivariable Cox were used to analyze the effects of patients’ clinicopathological factors on the predictive value of the risk score as an independent parameter. Although the advance clinical stage, TNM stage, and high score of risk were factors that made OS unfavorable, the most significant association was seen between the OS and the risk score in the multivariable Cox analysis (HR = 2.5700, *P* = 2.36e-06) (Table 5), indicating the independent prognostic value of IRG signatures—regardless of age, disease stage, and TNM stages—in LUAD patients.

**Table 5:** The univariate and multivariate cox regression analysis to evaluate the independent prognostic value

	**Unicox**			**Multicox**		
	**HR**	**95% CI of HR**	***P*-value**	**HR**	**95%CI of HR**	***P*-value**
**Age**	1.006	0.9908-1.021	0.456	1.0089	0.9908-1.027	0.3364
**Gender**	1.076	0.8071-1.435	0.618	0.9756	0.6966-1.366	0.8858
**Stage**	2.669	1.96-3.634	4.65e-10^***^	1.5022	0.9129-2.472	0.1093
**T**	2.285	1.562-3.345	2.1e-05^***^	1.7661	1.0910-2.859	0.0206^*^
**N**	2.521	1.883-3.375	5.18e-10^***^	1.6308	1.0938-2.431	0.0164^*^
**M**	2.198	1.284-3.761	0.00406^**^	1.2852	0.6640-2.488	0.4565
**Risk score**	2.837	2.076-3.877	5.94e-11^***^	2.5700	1.7366-3.803	2.36e-06^***^

^*^indicates a *P*-value < 0.05; ^**^indicates a *P*-value < 0.001; ^***^indicates a *P*-value < 0.0001

#### Association between the risk score and tumor-infiltrating immune cells

**Fig. 9:**
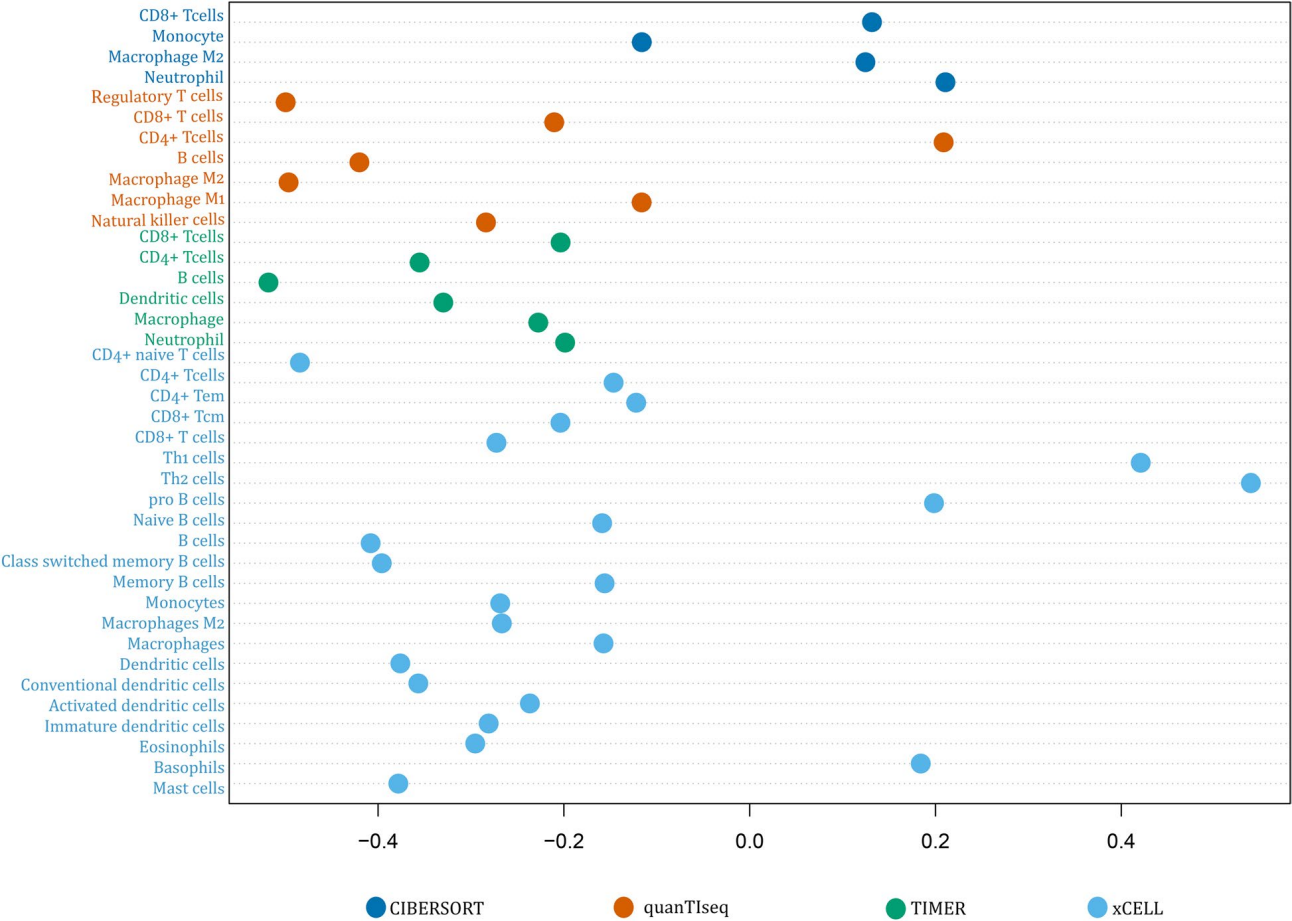
The correlation between risk score and tumor-infiltrating immune cells, which were analyzed by different quantification methods of immune infiltration estimations including CIBERSORT, quanTIseq, TIMER, XCell

Overall, the high-risk group demonstrated lower frequencies of immune cells. As represented in Fig. 9, not only DC and MQ but also T lymphocytes reduced in the tumor microenvironment of high-risk patients, suggesting that impaired antigen presentation to T cells may at least partly contribute to poor prognosis. Accordingly, NK cells—as the most important innate immune cells against cancer cells—were low in these patients. We also applied the Wilcoxon rank sum test on the results of XCELL and quanTIseq to investigate the association between the risk groups and tumor-infiltrating immune cells, depicted in Fig. S1.

#### Association between the risk score and mutation profile

**Fig. 10:**
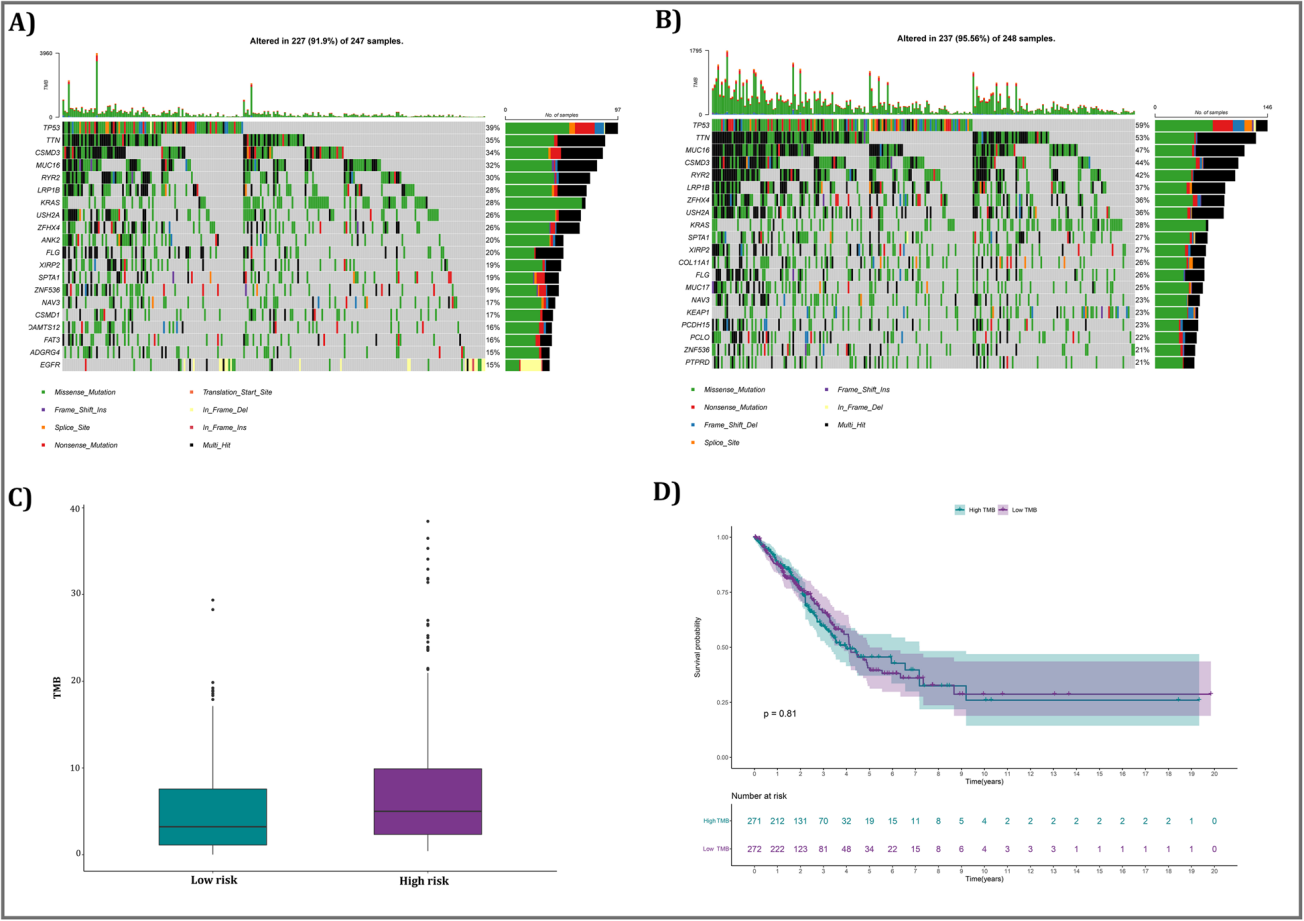
Tumor mutational burden (TMB) status among risk groups.** A** Mutation profile of the low-risk group. **B** Mutation profile of the high-risk group. **C** A correlation analysis between TMB and risk score. **D** The Kaplan–Meier curve analysis of high- and low-TMB groups

In the examination of LUAD patient mutation statuses, we have identified the top ten most significantly mutated genes for both high- and low-risk groups. These findings are visually represented in Fig. 10A and B. In the following analysis, we computed the TMB for each sample. Our findings revealed a considerably higher TMB in the high-risk patients (*P* = 3.148e-06) (Fig. 10C); however, we did not observe any relationship between TMB and OS (*P* = 0.81) (Fig. 10D). The results of this section will be further elaborated in the Discussion.

#### Association between the risk score and response to ICI

**Fig. 11:**
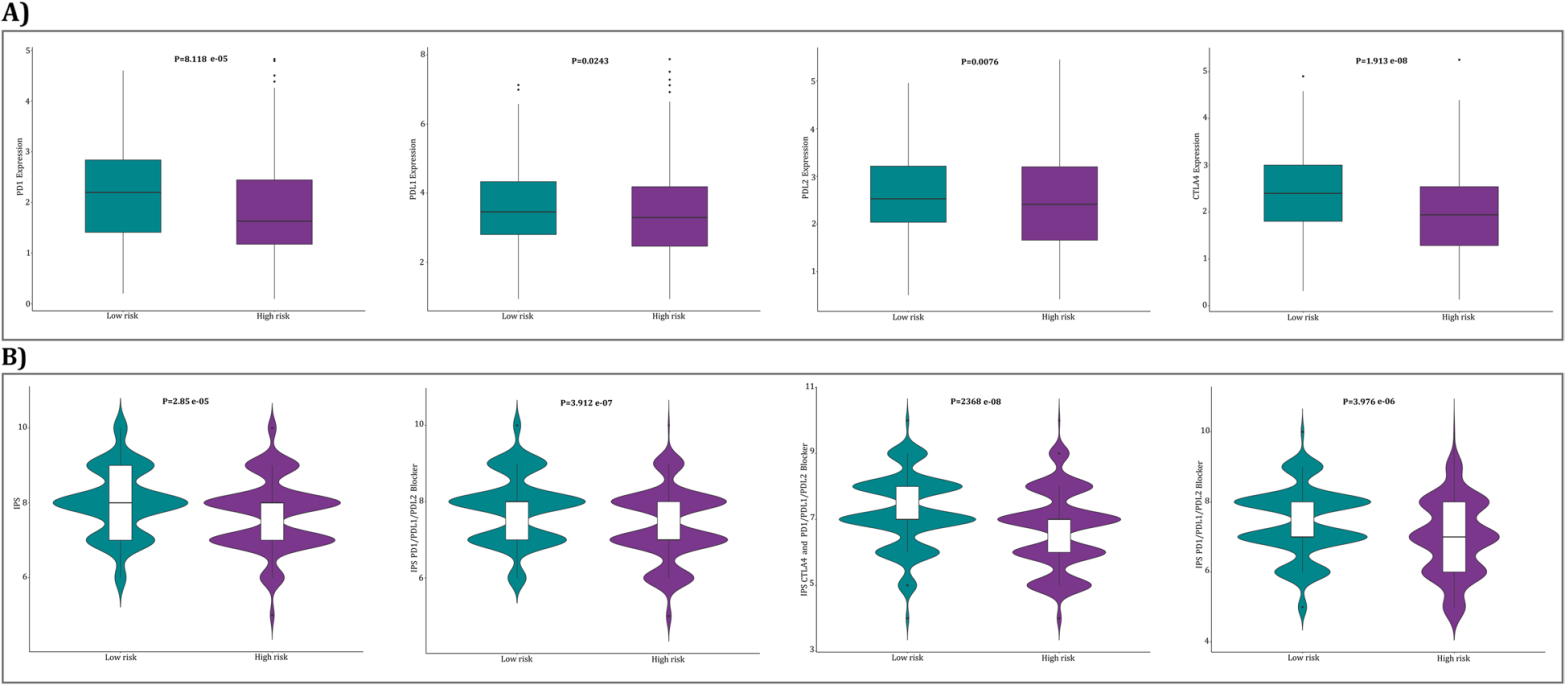
The association between risk groups and response to immune checkpoint inhibitors (ICI).** A** The gene expression of CTLA-4, PD-1, and PD-L1 in the high-risk and low-risk groups. **B** The association between IPS and the immune-related risk signature in LUAD patients

It has been confirmed that IPS could serve as predictive markers in melanoma patients undergoing treatment with PD-1 and CTLA-4 blockers [24]. Given this, it was tempting to investigate whether there is a relationship between our immune model and IPS. As represented in Fig. 11, the IPS scores exhibited a significant increase within the low-risk 9-IRG group, indicating a more pronounced immunogenic phenotype in this particular low-risk cohort. Furthermore, patients with a low risk had elevated levels of CTLA-4 (*P* = 1.913e-08), PD-1 (*P* = 8.118e-05), PDL-1 (*P* = 0.0243), and PDL-2 (*P* = 0.007663) expression, suggesting that ICI could be a promising treatment option for low-risk LUAD patients.

## Discussion

Lung cancer ranked as the primary cause of cancer-related fatalities in 2020, of which LUAD accounts for almost 40% [2]. Apart from environmental factors like occupational carcinogens, exposure to tobacco smoke, pre-existing non-malignant lung disease, and radon, molecular aberrations significantly influence the progression of lung cancer. In this regard, many signaling axes have been accused so far in the pathogenesis of this cancer; however, it appears that the mortality of lung cancer is caused by overlaps among these oncogenic pathways. According to the results of KEGG, we found that 91 DE-IRGs are mainly associated with several oncogenic pathways, such as PI3K-Akt, MAPK, RAS, and EGFR. Notably, the new wave of studies has uncovered the role played by the PI3K/Akt pathway not only in lung cancer cell survival but also at the crossroads of different cancer-related pathways [25]. The oncogenic role of MAPK, RAS, and EGFR deregulation has been also highlighted in the development of NSCLC [26]; interestingly, it has been indicated that up-regulation of CBLC—as one of the 9-IRG in our model—leads to enhanced stability of EGFR and sustained activation of its downstream signaling [27]. Given these, in recent years, PI3K, MAPK, and EGFR have been found to be viable therapeutic targets for novel treatments of cancer; however, lung cancer progression relies not only on the molecular features of tumor cells but also on their interaction with the tumor microenvironment, specifically with the immune cells [28]. In this vein, T cell activation-induced inhibitory checkpoint molecules, such as CTLA4, PD1, PDL1, and PDL2, are the most relevant target for immunotherapy nowadays [29], and certain ICIs are approved for the treatment of a wide range of malignancies including NSCLC [30].

Despite advances in ICI therapy, only a subset of patients achieves durable clinical benefits, and their survival rate is still unsatisfactory [4]. Accordingly, there is an urgent need to present specific biomarkers that can be used to assess risk and predict the prognosis of LUAD patients and facilitate the development of beneficial therapies. In the current investigation, we established a prognostic immune-related model by using 9-IRGs, which their details are summarized in Table 6. Four genes (BIRC5, CBLC, S100P, and LGR4) were associated with high risk, whereas five genes (SHC3, ANOS1, PGC, VIPR1, and IGKV4.1) were protective factors in LUAD patients. An increasing body of evidence supports the role of BIRC5, CBLC, VIPR1, and LGR4 in proliferation [31–34], as well as S100P and PGC in cancer metastasis [35, 36]. Interestingly, LGR4 alteration was associated with immunomodulation by promoting macrophage M2 polarization by Rspo/Lgr4/Erk/Stat3 signaling and restricting the anti-tumor activity of CD8^+^ T cells [37]. Notably, the infiltrating of immune cells into the TME contributes to different biological functions in malignancies, and the cross-talk between cancer and immune cells plays a pivotal role in determining the fate of tumor [38, 39].

**Table 6:** The details of 9-IRGs are included in the model

**Symbol**	**Protein**	**Alternation**	**Description**	**Alteration in other cancers**
BIRC5	Survivin	Up-regulated	• Inhibitor of apoptosis protein5 • Through its roles in mitosis, apoptosis suppression, autophagy, metabolism, angiogenesis, and migration, survivin can have multiple functions in promoting tumor cell survival and metastasis	Almost all cancers, such as pancreatic, breast, ovarian, brain, and colon cancer
CBLC	CBL proto-oncogene c	Up-regulated	• Enhanced stability of EGFR and sustained activation of its downstream signaling • Leads to uncontrolled cell proliferation, tumorigenesis, and cancer progression	Pancreas, breast, and colorectal carcinoma cells cancer
S100P	S100 calcium-binding protein P	Up-regulated	• Through combining Ca2 + ions, receptor for advanced glycation end products, cytoskeletal protein ezrin, calcyclin-binding protein/Siah-1-interacting protein, and cathepsin D, S100P plays a part in inducing tumor growth, metastasis, and invasion	Cervical, colon, breast, melanoma, ovarian, and oral cancer
IGKV4.1	Immunoglobulin kappa variable 4-1	Up-regulated	• IGKV4-1 gene encodes a B cell receptor	Breast, renal, head, and neck cancers
LGR4	Leucine-rich repeat-containing G-protein–coupled receptor 4	Up-regulated	• Lgr4 and its ligands R-spondin have been shown to promote the growth and metastasis of tumor cells • Promotes macrophage M2 polarization through Rspo/Lgr4/Erk/Stat3 signaling	Multiple myeloma, thyroid carcinoma, ovarian, prostate, colon, and breast cancer
PGC	Pepsinogen C	Down-regulated	• PGC in the acidic organelles hydrolyses pro-surfactant protein B (pro-SPB), which is secreted by alveolar type 2 epithelial cells. So, it plays a major role in lung maturation	Gastric, breast, prostate, ovarian, endometrial, pancreatic, kidney, bladder, squamous cell carcinoma, and melanoma cancers
SHC3	SHC adaptor protein 3	Down-regulated	• Signaling adapter that couples activated growth factor receptors to signaling pathway in neurons	
ANOS1	Anosmin-1	Down-regulated	• Anosmin-1 is an extracellular matrix protein with adhesion and chemoattractant characteristics	Colon, ovary, hepatocellular carcinoma, breast cancer
VIPR1	Vasoactive Intestinal Peptide receptor	Down-regulated	• A receptor for vasoactive intestinal peptide (VIP), a small neuropeptide • VIPR1 inhibits the growth, migration, and invasion of several cancers	Hepatocellular carcinoma

**Fig. 12:**
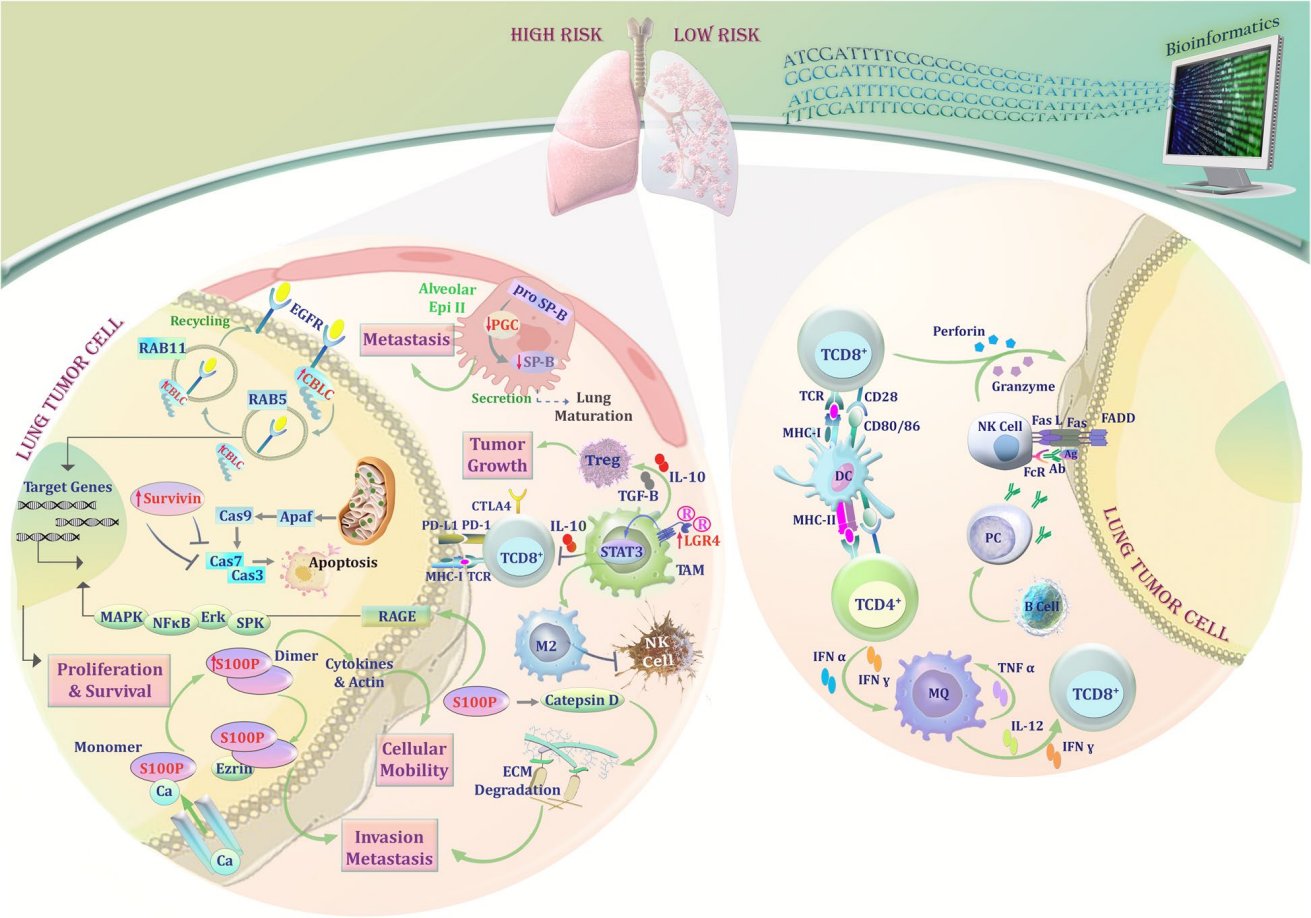
A plausible schematic of underlying mechanisms of CBLC, BIRC5, S100P, PGC, and LGR4 genes with a glance at the tumor immune microenvironment of high- and low-risk groups. The upregulated CBLC mediates polyubiquitination of EGFR and promotes its trafficking into the nucleus or recycling back to the cell membrane, leading to enhanced stability of EGFR and sustained activation of its downstream signaling. BIRC5 (survivin) binds and suppresses effector caspases, resulting in decreased apoptosis. The S100P protein is expressed in an inactive state and triggered by calcium ions to form active dimers; they can operate intracellularly or as extracellular signaling molecules. Inside the cell, binding of S100P to ezrin leads to its activation, followed by the regulation of invasion and metastasis. The secreted form of S100P can bind to the extracellular ligand-binding site of RAGE and, via activation of the ERK/MAPK pathway, influences gene expression. Downregulation of PGC inhibits pro-surfactant protein B (pro-SPB) maturation, resulting in tumor cell dedifferentiation or deterioration, closely related to cancer metastasis. LGR4 promotes macrophage M2 polarization by Rspo/Lgr4/Erk/Stat3 signaling and restricting the anti-tumor activity of CD8^+^ T cells and NK cells. The tumor microenvironment of low-risk patients contains effector cells like CD8^+^ T cells and NK cells. On the other hand, the tumor microenvironment of high-risk patients is suppressed by immunosuppressor cells such as macrophage M2 and Treg

For further investigation, we applied several algorithms to assess the status of immune infiltration in both low-risk and high-risk cohorts. Our findings revealed a negative correlation between the risk scores of LUAD patients and the presence of immune cells within the tumor; it appears that according to the low frequency of DC, MQ, and different types of T cells in high-risk patients, antigen presentation, T cell activation, and finally, killing of cancer cells are hampered in these patients. Notably, it has been documented that CD8^+^ T cell infiltration in the TME is associated with improved cancer patient responses to ICIs; Wong et al. demonstrated that melanoma patients who received anti-PD-1 therapy experienced prolonged survival when they had a high CD8^+^ T cell count [40]. Figure 12 provides a better overview of the TIME and underlying mechanisms of our 9-IRGs.

Apart from immune cell infiltration, it is reported that TMB could be a possible predictive factor for ICI therapy. A recent meta-analysis containing 11 studies demonstrated that NSCLC patients with high TMB could benefit more from immunotherapy than patients with low TMB [41]; however, several other studies showed that high TMB failed to predict ICI response across all cancer types [42–44]. There is also a controversy about the cutoff value of the TMB [45]. In alignment with a prior investigation, we have observed a notable decrease in the TMB within the low-risk patients [46], indicating that high TMB does not necessarily lead to a better response to ICI therapy. The rationale for this could be that the IPS is a multifaceted model comprising various variables. As a result, it is feasible that other elements, such as increased expression of immune checkpoints, might contribute to a better ICI response in the low-risk cohort.

Since it has been proved that IPS has a predictive value in patients receiving PD-1 and CTLA-4 inhibitors for melanoma [24], we investigated IPS among our risk groups. According to the results, low-risk patients had significantly higher IPS values, meaning that the immunogenicity of the tumor immune contexture was also elevated in this group. To reconfirm the checkpoint inhibitor-based immunotherapy efficacy in LUAD samples with different risk scores, we also investigated the expression of immune checkpoint genes. The findings revealed that the low-risk group had high levels of their expression, indirectly implying the preexisted T cell activation for this group, suggesting that they had a better chance of receiving ICI treatment.

## Conclusion

Taken together, we developed an IRG-based prognostic model in LUAD patients, which is predictive of patients’ survival and ICI immunotherapy outcomes and reflects the tumor immune microenvironment status based on RNA sequencing data. We believe that this signature might be helpful in managing LUAD patients in clinical practice; however, its validation in clinical settings is required.

## Supplementary Information

Supplementary Material 1.

## Data Availability

The datasets used and/or analyzed during the current study are available from the corresponding author on reasonable request.

## Abbreviations

ICI: Immune checkpoint inhibitor
LUAD: Lung adenocarcinoma
IPS: Immunophenoscore
IRGs: Immune-related genes
OS: Overall survival
TMB: The Tumor Mutation Burden
NSCLC: Non-small-cell lung cancer
TME: Tumor immune microenvironment
ECM: Extracellular matrix
TCGA: The Cancer Genome Atlas Lung Adenocarcinoma
GEO: Gene Expression Omnibus
ImmPort: Immunology Database And Analysis Portal
TCIA: The Cancer Immunome Atlas
DEGs: Differentially expressed genes
GO: Gene Ontology
KEGG: Kyoto Encyclopedia Of Genes And Genomes
ROC: Receiver operating characteristic
AUC: Area under the curve
MAF: Mutation annotation format
HR: Hazard ratio
