# Supplementary material for: Identification of an immune-related genes signature in lung adenocarcinoma to predict survival and response to immune checkpoint inhibitors
Source: J Egypt Natl Canc Inst. 2024 Oct 7;36:30. doi: 10.1186/s43046-024-00236-0 (PMC13313863; doi:10.1186/s43046-024-00236-0)
Supplement: Supplementary file 1 — Supplementary Material 1. [file 43046_2024_236_MOESM1_ESM.docx]

**Table S1:** Clinical characteristics of patients with LUAD in total, training and test cohorts.

|  | **Total cohort (n=526)** | **Training cohort (n=421)** | **Test cohort (n=105)** | **P.value** | **Method** |
| --- | --- | --- | --- | --- | --- |
| **Survival time (days)** | 913.6±38.35* | 933.1±44.2 | 835.4±74.06 | 0.2591 | t-test |
| **Vital status** | | | | | |
| Alive | 339 (64.44%) | 275 (65.32%) | 64 (60.95%) | 0.8617 | X^2^ test |
| Dead | 187 (35.55%) | 146 (34.67%) | 41 (39.04%) |  |  |
| **Age** | | | | | |
| ≤65 | 256 (48.66%) | 205 (48.69%) | 51 (48.57%) | 0.3569 | Fisher’s exact test |
| >65 | 260 (49.42%) | 206(48.93%) | 54 (51.42%) |  |  |
| Unknown | 10 (1.90%) | 10 (2.37%) | - |  |  |
| **Gender** | | | | | |
| Female | 283 (53.80%) | 226 (53.68%) | 57 (54.28%) | 0.9987 | X^2^ test |
| Male | 243 (46.19%) | 195 (46.31%) | 48 (45.71%) |  |  |
| **Stage** | | | | | |
| I | 290 (55.13%) | 232 (55.10%) | 58 (55.23%) | 0.6315 | X^2^ test |
| II | 123 (23.38%) | 98 (23.27%) | 25 (23.80%) |  |  |
| III | 80 (15.20%) | 62 (14.72%) | 18 (17.14%) |  |  |
| IV | 25 (4.75%) | 21 (4.98%) | 4 (3.80%) |  |  |
| Unknown | 8 (1.52%) | 8 (1.90%) | - |  |  |
| **T category** | | | | | |
| T1 | 175 (33.26%) | 140 (33.25%) | 35 (33.33%) | 0.07637 | X^2^ test |
| T2 | 283 (53.80%) | 235 (55.81%) | 48 (45.71%) |  |  |
| T3 | 47 (8.93%) | 31 (7.36%) | 16 (15.23%) |  |  |
| T4 | 18 (3.42%) | 13 (3.08%) | 5 (4.76%) |  |  |
| TX | 3 (0.57%) | 2 (0.47%) | 1 (0.95%) |  |  |
| **N category** | | | | | |
| N0 | 344 (65.39%) | 273 (64.84%) | 71 (67.61%) | 0.9493 | X^2^ test |
| N1 | 96 (18.25%) | 77 (18.28%) | 19 (18.09%) |  |  |
| N2 | 69 (13.11%) | 57 (13.53%) | 12 (11.42%) |  |  |
| N3 | 2 (0.38%) | 2 (0.47%) | - |  |  |
| NX | 14 (2.66%) | 11 (2.61%) | 3 (2.85%) |  |  |
| Unknown | 1 (0.19%) | 1 (0.23%) | - |  |  |
| **M category** | | | | | |
| M0 | 353 (67.11%) | 283 (67.22%) | 70 (66.66%) | 0.96 | X^2^ test |
| M1 | 24 (4.56%) | 20 (4.75%) | 4 (3.80%) |  |  |
| MX | 143 (27.18%) | 113 (26.84%) | 30 (28.57%) |  |  |
| Unknown | 6 (1.14%) | 5 (1.18%) | 1 (0.95%) |  |  |
| *Mean ± SE (standard error).  **TX**: Primary tumor could not be assessed; **NX**: Regional lymph nodes could not be assessed; **MX**: Distant metastases could not be assessed. | | | | | |

**Table S2:** 91 differentially expressed IRGs between LUAD and normal samples.

|  | **Symbol** | **logFC** | **AveExpr** | **t** | **P.Value** | **adj.P.Val** | **B** | **Type** |
| --- | --- | --- | --- | --- | --- | --- | --- | --- |
| 1 | CRABP2 | 4.037136 | 5.794837 | 13.3486 | 9.19E-36 | 1.95E-34 | 70.18251 | Up regulated |
| 2 | IGHG4 | 3.098369 | 6.997614 | 10.31123 | 4.67E-23 | 5.12E-22 | 41.14474 | Up regulated |
| 3 | BIRC5 | 2.871818 | 4.594143 | 15.15705 | 3.73E-44 | 1.23E-42 | 89.39831 | Up regulated |
| 4 | S100P | 2.765808 | 5.282677 | 6.97304 | 8.23E-12 | 4.04E-11 | 15.59521 | Up regulated |
| 5 | MMP12 | 2.749454 | 3.707234 | 10.12639 | 2.37E-22 | 2.5E-21 | 39.53725 | Up regulated |
| 6 | IGHG1 | 2.626427 | 11.10513 | 8.853512 | 9.58E-18 | 7.44E-17 | 29.04679 | Up regulated |
| 7 | PAEP | 2.470389 | 2.679022 | 6.377013 | 3.61E-10 | 1.52E-09 | 11.89367 | Up regulated |
| 8 | CXCL13 | 2.464628 | 3.929977 | 9.852717 | 2.52E-21 | 2.49E-20 | 37.19509 | Up regulated |
| 9 | IGHG2 | 2.403258 | 8.443296 | 8.744776 | 2.26E-17 | 1.72E-16 | 28.19989 | Up regulated |
| 10 | CBLC | 2.306817 | 3.806044 | 14.33236 | 2.9E-40 | 7.81E-39 | 80.48719 | Up regulated |
| 11 | LGR4 | 2.303644 | 5.7112 | 16.42222 | 2.67E-50 | 1.16E-48 | 103.4833 | Up regulated |
| 12 | IGHG3 | 2.236123 | 8.479978 | 7.969851 | 8.07E-15 | 5.11E-14 | 22.40335 | Up regulated |
| 13 | CXCL14 | 2.186701 | 4.832779 | 6.462637 | 2.14E-10 | 9.18E-10 | 12.40784 | Up regulated |
| 14 | PLXNB3 | 2.166909 | 3.508852 | 10.32958 | 3.97E-23 | 4.37E-22 | 41.30543 | Up regulated |
| 15 | IGKC | 2.148665 | 11.26387 | 7.998314 | 6.55E-15 | 4.17E-14 | 22.60867 | Up regulated |
| 16 | IGKV4-1 | 2.140969 | 6.689269 | 7.596578 | 1.18E-13 | 6.78E-13 | 19.76599 | Up regulated |
| 17 | IGKV3-15 | 2.105894 | 5.263767 | 8.090497 | 3.32E-15 | 2.17E-14 | 23.27767 | Up regulated |
| 18 | IGHV1-24 | 2.060966 | 3.891342 | 7.486733 | 2.54E-13 | 1.42E-12 | 19.00968 | Up regulated |
| 19 | IGLV3-21 | 2.034292 | 5.980241 | 7.134217 | 2.82E-12 | 1.45E-11 | 16.64458 | Up regulated |
| 20 | HNF4G | 2.019592 | 2.639255 | 11.78955 | 5.42E-29 | 8.24E-28 | 54.69776 | Up regulated |
| 21 | GPER1 | -2.00649 | 1.970201 | -15.9551 | 5.22E-48 | 2.07E-46 | 98.22965 | Down regulated |
| 22 | IL20RA | -2.01281 | 3.018187 | -11.5865 | 3.79E-28 | 5.49E-27 | 52.76685 | Down regulated |
| 23 | NPR3 | -2.01413 | 4.18926 | -12.4158 | 1.18E-31 | 2.07E-30 | 60.78265 | Down regulated |
| 24 | CXCR2 | -2.03277 | 1.443833 | -18.3627 | 4.63E-60 | 3.08E-58 | 125.8638 | Down regulated |
| 25 | SLC11A1 | -2.05436 | 4.924725 | -13.8337 | 5.81E-38 | 1.38E-36 | 75.21645 | Down regulated |
| 26 | FGF2 | -2.06697 | 2.134399 | -16.6418 | 2.19E-51 | 1.01E-49 | 105.9726 | Down regulated |
| 27 | FABP5 | -2.06877 | 3.057662 | -14.5546 | 2.65E-41 | 7.5E-40 | 82.86559 | Down regulated |
| 28 | ROBO2 | -2.11531 | 2.704595 | -11.7268 | 9.9E-29 | 1.48E-27 | 54.09917 | Down regulated |
| 29 | RXRG | -2.13882 | 1.055773 | -17.6648 | 1.64E-56 | 9.71E-55 | 117.7222 | Down regulated |
| 30 | IL6 | -2.1437 | 2.481378 | -11.4177 | 1.88E-27 | 2.64E-26 | 51.17704 | Down regulated |
| 31 | SHC3 | -2.14838 | 3.137698 | -13.4471 | 3.31E-36 | 7.22E-35 | 71.19722 | Down regulated |
| 32 | C5AR1 | -2.15247 | 4.674747 | -15.5353 | 5.7E-46 | 2.05E-44 | 93.55961 | Down regulated |
| 33 | TIE1 | -2.15579 | 4.738419 | -17.9365 | 6.88E-58 | 4.28E-56 | 120.881 | Down regulated |
| 34 | NR4A1 | -2.16217 | 6.185557 | -10.173 | 1.58E-22 | 1.68E-21 | 39.94085 | Down regulated |
| 35 | BMP2 | -2.17784 | 4.363404 | -11.8583 | 2.79E-29 | 4.3E-28 | 55.35596 | Down regulated |
| 36 | EDN1 | -2.1813 | 4.4628 | -12.6544 | 1.09E-32 | 2E-31 | 63.14991 | Down regulated |
| 37 | CXCL2 | -2.18212 | 4.782122 | -10.81 | 5.31E-25 | 6.43E-24 | 45.58234 | Down regulated |
| 38 | COLEC12 | -2.21474 | 5.197827 | -12.5553 | 2.94E-32 | 5.29E-31 | 62.16348 | Down regulated |
| 39 | FGF10 | -2.24001 | 0.860971 | -24.5216 | 1.94E-92 | 5.58E-90 | 200.1472 | Down regulated |
| 40 | A2M | -2.25378 | 9.820135 | -14.6218 | 1.28E-41 | 3.7E-40 | 83.58769 | Down regulated |
| 41 | FGR | -2.28788 | 4.454021 | -16.8593 | 1.82E-52 | 8.94E-51 | 108.4501 | Down regulated |
| 42 | AHNAK | -2.28827 | 9.234125 | -14.7074 | 5.08E-42 | 1.49E-40 | 84.51034 | Down regulated |
| 43 | IL7R | -2.3025 | 5.658169 | -13.3898 | 6E-36 | 1.29E-34 | 70.60612 | Down regulated |
| 44 | LEPR | -2.30947 | 4.183128 | -15.0524 | 1.18E-43 | 3.75E-42 | 88.2544 | Down regulated |
| 45 | FGFR2 | -2.31659 | 4.433234 | -13.2684 | 2.1E-35 | 4.42E-34 | 69.35936 | Down regulated |
| 46 | SEMA3B | -2.31959 | 4.761709 | -11.1731 | 1.87E-26 | 2.45E-25 | 48.90111 | Down regulated |
| 47 | ANGPTL1 | -2.31969 | 1.779911 | -18.8368 | 1.7E-62 | 1.3E-60 | 131.4454 | Down regulated |
| 48 | NR4A3 | -2.32941 | 3.3959 | -12.0956 | 2.78E-30 | 4.51E-29 | 57.64717 | Down regulated |
| 49 | SEMA6D | -2.35704 | 2.503944 | -16.2064 | 3.08E-49 | 1.29E-47 | 101.0482 | Down regulated |
| 50 | RXFP1 | -2.46025 | 1.265283 | -26.5171 | 4.9E-103 | 2.2E-100 | 224.4743 | Down regulated |
| 51 | SLPI | -2.49697 | 7.477119 | -8.99384 | 3.13E-18 | 2.52E-17 | 30.15157 | Down regulated |
| 52 | NOS1 | -2.5321 | 1.182442 | -15.5278 | 6.2E-46 | 2.23E-44 | 93.4766 | Down regulated |
| 53 | ANGPT4 | -2.5487 | 0.693557 | -36.0311 | 5.3E-152 | 1.8E-148 | 336.7794 | Down regulated |
| 54 | MASP1 | -2.57743 | 1.802162 | -20.4547 | 6.64E-71 | 6.72E-69 | 150.7362 | Down regulated |
| 55 | ANGPT1 | -2.58082 | 3.478574 | -16.1523 | 5.67E-49 | 2.35E-47 | 100.4405 | Down regulated |
| 56 | SSTR1 | -2.60397 | 1.937435 | -15.6125 | 2.41E-46 | 8.83E-45 | 94.41442 | Down regulated |
| 57 | SEMA6A | -2.65617 | 3.143102 | -20.358 | 2.13E-70 | 2.11E-68 | 149.5738 | Down regulated |
| 58 | IL33 | -2.66614 | 4.466273 | -14.6055 | 1.53E-41 | 4.38E-40 | 83.41298 | Down regulated |
| 59 | ACKR1 | -2.66913 | 3.384895 | -13.3982 | 5.5E-36 | 1.19E-34 | 70.6933 | Down regulated |
| 60 | FPR2 | -2.67037 | 1.894889 | -18.7451 | 5.05E-62 | 3.76E-60 | 130.3629 | Down regulated |
| 61 | ACVRL1 | -2.67365 | 5.146292 | -24.3143 | 2.46E-91 | 6.51E-89 | 197.616 | Down regulated |
| 62 | MSR1 | -2.68212 | 5.778063 | -15.3282 | 5.66E-45 | 1.92E-43 | 91.27554 | Down regulated |
| 63 | SLIT2 | -2.69657 | 4.630457 | -18.5143 | 7.74E-61 | 5.4E-59 | 127.644 | Down regulated |
| 64 | RBP4 | -2.72808 | 2.283917 | -14.4766 | 6.15E-41 | 1.72E-39 | 82.02913 | Down regulated |
| 65 | DMBT1 | -2.87851 | 5.689712 | -7.86582 | 1.72E-14 | 1.06E-13 | 21.65798 | Down regulated |
| 66 | TGFBR3 | -2.88918 | 3.988069 | -19.5057 | 5.93E-66 | 5.06E-64 | 139.3791 | Down regulated |
| 67 | CALCRL | -2.89492 | 5.129118 | -21.4172 | 5.77E-76 | 7.04E-74 | 162.3487 | Down regulated |
| 68 | SEMA5A | -2.92407 | 4.333222 | -18.015 | 2.75E-58 | 1.72E-56 | 121.7967 | Down regulated |
| 69 | S1PR1 | -2.95705 | 4.699227 | -24.3786 | 1.12E-91 | 3.09E-89 | 198.4008 | Down regulated |
| 70 | OLR1 | -2.97918 | 4.890469 | -15.9841 | 3.77E-48 | 1.51E-46 | 98.55436 | Down regulated |
| 71 | NPR1 | -3.00157 | 3.70212 | -19.8391 | 1.09E-67 | 9.88E-66 | 143.3566 | Down regulated |
| 72 | SEMA3G | -3.00718 | 3.298812 | -23.7102 | 4.02E-88 | 8.66E-86 | 190.2436 | Down regulated |
| 73 | FGFR4 | -3.0156 | 3.61562 | -15.9757 | 4.14E-48 | 1.66E-46 | 98.45997 | Down regulated |
| 74 | IL1RL1 | -3.06117 | 2.265098 | -15.3556 | 4.18E-45 | 1.43E-43 | 91.57765 | Down regulated |
| 75 | ADRB2 | -3.17191 | 2.700207 | -23.418 | 1.44E-86 | 3E-84 | 186.6789 | Down regulated |
| 76 | ADRB1 | -3.21793 | 2.021941 | -20.2446 | 8.36E-70 | 8.01E-68 | 148.2128 | Down regulated |
| 77 | ANOS1 | -3.22161 | 4.942725 | -18.6669 | 1.28E-61 | 9.36E-60 | 129.4409 | Down regulated |
| 78 | VIPR1 | -3.22612 | 3.760437 | -18.9686 | 3.57E-63 | 2.77E-61 | 133.0029 | Down regulated |
| 79 | GDF10 | -3.35949 | 1.870528 | -21.8015 | 5.39E-78 | 7.35E-76 | 167.0055 | Down regulated |
| 80 | TEK | -3.36561 | 3.638512 | -25.682 | 1.31E-98 | 5.16E-96 | 214.3043 | Down regulated |
| 81 | AGTR2 | -3.3808 | 1.852025 | -15.39 | 2.85E-45 | 9.88E-44 | 91.95638 | Down regulated |
| 82 | DUOX1 | -3.44407 | 4.910868 | -15.9699 | 4.43E-48 | 1.76E-46 | 98.39448 | Down regulated |
| 83 | DES | -3.5051 | 2.912256 | -18.1304 | 7.09E-59 | 4.56E-57 | 123.1443 | Down regulated |
| 84 | PGC | -3.56637 | 5.648728 | -7.26368 | 1.18E-12 | 6.25E-12 | 17.50205 | Down regulated |
| 85 | EDNRB | -3.76486 | 4.050195 | -24.459 | 4.18E-92 | 1.18E-89 | 199.3823 | Down regulated |
| 86 | SFTPD | -3.7896 | 6.359749 | -12.1454 | 1.7E-30 | 2.8E-29 | 58.13207 | Down regulated |
| 87 | MARCO | -3.96197 | 5.261889 | -15.6977 | 9.33E-47 | 3.48E-45 | 95.36055 | Down regulated |
| 88 | SFTPA2 | -4.85452 | 9.991271 | -11.1716 | 1.89E-26 | 2.48E-25 | 48.88749 | Down regulated |
| 89 | SFTPA1 | -5.06435 | 9.573952 | -11.4442 | 1.46E-27 | 2.06E-26 | 51.42637 | Down regulated |
| 90 | FABP4 | -5.3255 | 2.303883 | -29.1157 | 9.7E-117 | 6.6E-114 | 255.9093 | Down regulated |
| 91 | AGER | -6.59504 | 5.264909 | -24.1156 | 2.81E-90 | 6.95E-88 | 195.1906 | Down regulated |

**Table S3:** ToppFun Enrichment.

| **ID** | **Name** | **P-value** | **FDR B&H** | **FDR B&Y** | **Bonferroni** | **Input genes** |
| --- | --- | --- | --- | --- | --- | --- |
| **GO: Molecular Function** | | | | | | |
| GO:0005102 | Signaling receptor binding | 1.177E-17 | 4.873E-15 | 3.218E-14 | 4.873E-15 | 39 |
| GO:0004888 | Transmembrane signaling receptor activity | 7.579E-17 | 1.569E-14 | 1.036E-13 | 3.138E-14 | 34 |
| GO:0034988 | Fc-gamma receptor I complex binding | 3.672E-11 | 5.067E-9 | 3.346E-8 | 1.520E-8 | 5 |
| GO:0030545 | Signaling receptor regulator activity | 1.809E-10 | 1.873E-8 | 1.237E-7 | 7.490E-8 | 18 |
| GO:0030546 | Signaling receptor activator activity | 3.701E-10 | 3.065E-8 | 2.024E-7 | 1.532E-7 | 17 |
| **GO: Biological Process** | | | | | | |
| GO:0032101 | Regulation of response to external stimulus | 1.921E-20 | 7.723E-17 | 6.856E-16 | 7.723E-17 | 36 |
| GO:0040011 | Locomotion | 6.044E-19 | 1.173E-15 | 1.041E-14 | 2.430E-15 | 40 |
| GO:0006935 | Chemotaxis | 1.179E-18 | 1.173E-15 | 1.041E-14 | 4.740E-15 | 29 |
| GO:0042330 | Taxis | 1.257E-18 | 1.173E-15 | 1.041E-14 | 5.054E-15 | 29 |
| GO:0035295 | Tube development | 1.458E-18 | 1.173E-15 | 1.041E-14 | 5.863E-15 | 40 |
| **Go: Cellular Component** | | | | | | |
| GO:0009986 | Cell surface | 5.424E-14 | 1.416E-11 | 8.697E-11 | 1.416E-11 | 28 |
| GO:0031226 | Intrinsic component of plasma membrane | 1.187E-13 | 1.431E-11 | 8.790E-11 | 3.098E-11 | 35 |
| GO:0005887 | Integral component of plasma membrane | 1.645E-13 | 1.431E-11 | 8.790E-11 | 4.292E-11 | 34 |
| GO:0031012 | Extracellular matrix | 1.015E-12 | 5.606E-11 | 3.444E-10 | 2.650E-10 | 21 |
| GO:0030312 | External encapsulating structure | 1.074E-12 | 5.606E-11 | 3.444E-10 | 2.803E-10 | 21 |
| **Pathway** | | | | | | |
| M5885 | Ensemble of genes encoding ECM-associated proteins including ECM-affilaited proteins, ECM regulators and secreted factors | 2.100E-13 | 1.890E-10 | 1.395E-9 | 1.890E-10 | 26 |
| M5889 | Ensemble of genes encoding extracellular matrix and extracellular matrix-associated proteins | 9.879E-13 | 4.445E-10 | 3.281E-9 | 8.891E-10 | 29 |
| 1269545 | Class A/1 (Rhodopsin-like receptors) | 9.995E-10 | 2.998E-7 | 2.213E-6 | 8.995E-7 | 15 |
| 1269546 | Peptide ligand-binding receptors | 1.613E-9 | 3.630E-7 | 2.679E-6 | 1.452E-6 | 12 |
| 1269243 | Creation of C4 and C2 activators | 2.324E-9 | 4.184E-7 | 3.088E-6 | 2.092E-6 | 6 |
| **PubMed** | | | | | | |
| 24952961 | A high-resolution spatiotemporal atlas of gene expression of the developing mouse brain. | 5.509E-21 | 2.083E-16 | 2.316E-15 | 2.083E-16 | 32 |
| 29031500 | Transcription and Signaling Regulators in Developing Neuronal Subtypes of Mouse and Human Enteric Nervous System. | 1.536E-16 | 2.903E-12 | 3.228E-11 | 5.806E-12 | 14 |
| 7704573 | Cre-loxP-mediated gene replacement: a mouse strain producing humanized antibodies | 3.113E-14 | 2.354E-10 | 2.617E-9 | 1.177E-9 | 5 |
| 103631 | The synthesis and processing of the mRNAs specifying heavy and light chain immunoglobulins in MPC-11 cells | 3.113E-14 | 2.354E-10 | 2.617E-9 | 1.177E-9 | 5 |
| 5073237 | The disulphide bridges of a mouse immunoglobulin G1 protein. | 3.113E-14 | 2.354E-10 | 2.617E-9 | 1.177E-9 | 5 |
| **Interaction** | | | | | | |
| int:SFTPA1 | SFTPA1 interactions | 2.703E-8 | 9.559E-5 | 8.363E-4 | 9.559E-5 | 4 |
| int:FGF2 | FGF2 interactions | 9.799E-8 | 1.733E-4 | 1.516E-3 | 3.466E-4 | 6 |
| int:UGT1A10 | UGT1A10 interactions | 3.096E-7 | 3.650E-4 | 3.193E-3 | 1.095E-3 | 5 |
| int:TEK | TEK interactions | 4.247E-7 | 3.755E-4 | 3.285E-3 | 1.502E-3 | 5 |
| int:CIR1 | CIR1 interactions | 5.787E-7 | 4.029E-4 | 3.525E-3 | 2.047E-3 | 6 |
| **Gene Family** | | | | | | |
| 736 | Immunoglobulin like domain containing\|Semaphorins | 3.360E-8 | 2.150E-6 | 1.020E-5 | 2.150E-6 | 5 |
| 491 | Collectins | 1.026E-7 | 3.284E-6 | 1.558E-5 | 6.569E-6 | 4 |
| 1253 | Scavenger receptors | 1.704E-7 | 3.635E-6 | 1.724E-5 | 1.090E-5 | 5 |
| 471 | CD molecules\|C-type lectin domain family | 1.933E-5 | 3.093E-4 | 1.467E-3 | 1.237E-3 | 10 |
| 321 | Receptor Tyrosine Kinases\|CD molecules\|Immunoglobulin like domain containing | 4.001E-5 | 3.892E-4 | 1.846E-3 | 2.561E-3 | 4 |
| **Drug** | | | | | | |
| ctd:D010126 | Ozone | 5.621E-20 | 9.181E-16 | 9.437E-15 | 9.181E-16 | 34 |
| ctd:C007350 | Nitrofen | 5.290E-17 | 4.320E-13 | 4.440E-12 | 8.640E-13 | 17 |
| ctd:D002713 | Chlorine | 7.429E-14 | 3.923E-10 | 4.032E-9 | 1.213E-9 | 17 |
| ctd:D019821 | Simvastatin | 9.608E-14 | 3.923E-10 | 4.032E-9 | 1.569E-9 | 20 |
| CID000000158 | 8-iso-prostaglandin E2 | 1.915E-13 | 6.255E-10 | 6.429E-9 | 3.127E-9 | 20 |
| **Disease** | | | | | | |
| C0024117 | Chronic Obstructive Airway Disease | 1.567E-22 | 7.650E-19 | 6.939E-18 | 7.650E-19 | 38 |
| C0007222 | Cardiovascular Diseases | 9.567E-21 | 2.336E-17 | 2.119E-16 | 4.672E-17 | 40 |
| C0003850 | Arteriosclerosis | 1.658E-19 | 2.144E-16 | 1.945E-15 | 8.097E-16 | 43 |
| C0004153 | Atherosclerosis | 1.756E-19 | 2.144E-16 | 1.945E-15 | 8.576E-16 | 43 |
| C0004096 | Asthma | 4.088E-18 | 3.992E-15 | 3.621E-14 | 1.996E-14 | 39 |


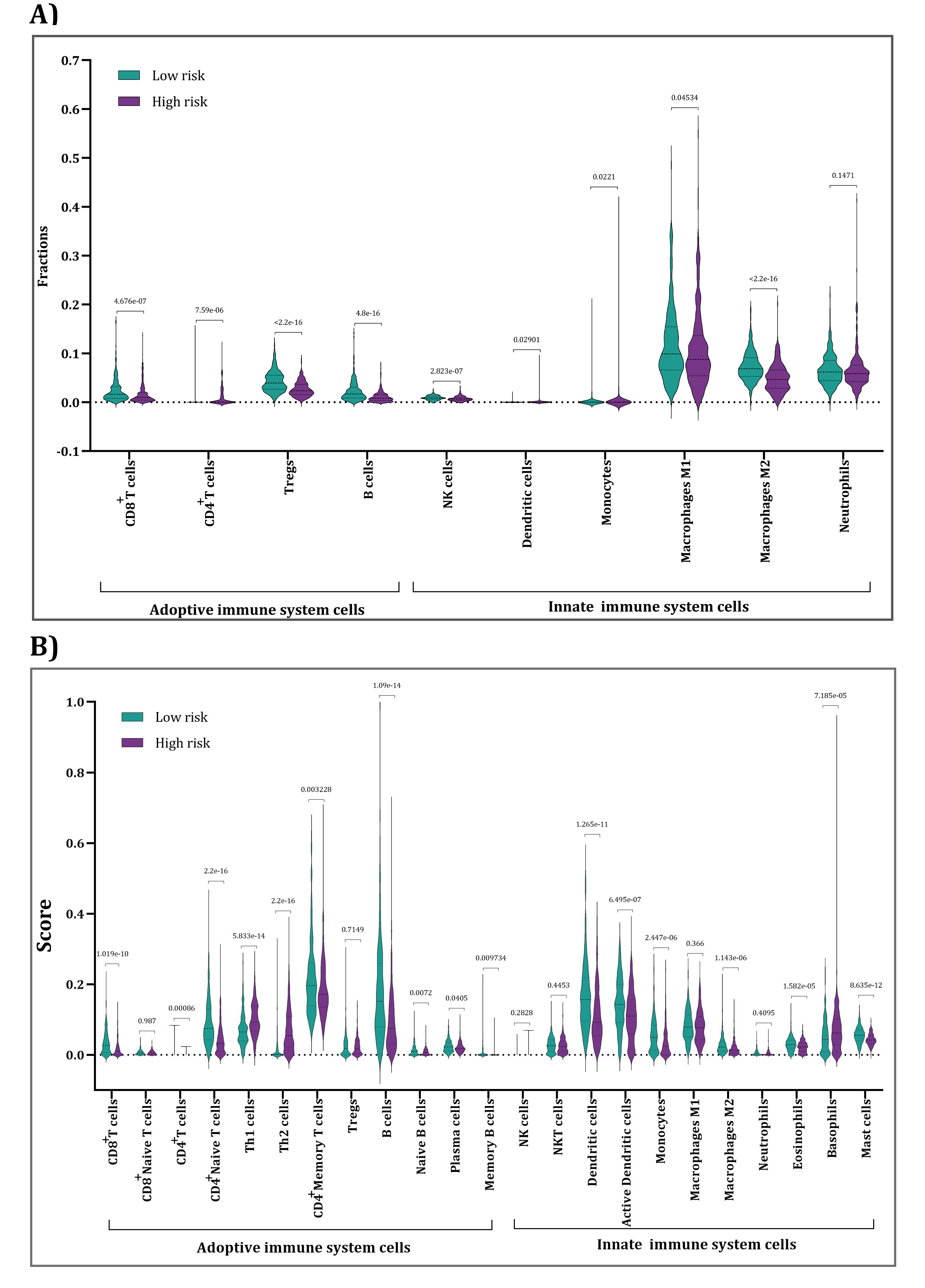


**Figure S1. The association of immune cell infiltration and the immune-related risk signature in LUAD patients,** which were analyzed by **A)** quanTIseq and **B)** XCELL methods.
